# Takotsubo Cardiomyopathy Triggered by Acute Intermittent Porphyria

**DOI:** 10.7759/cureus.41185

**Published:** 2023-06-30

**Authors:** Talal Alzahrani

**Affiliations:** 1 Internal Medicine, Taibah University - College of Medicine, Madinah, SAU

**Keywords:** management, stress induced cardiomyopathy, clinical outcome, takotsubo cardiomyopathy, porphyria

## Abstract

Background

Takotsubo cardiomyopathy (TC) is a reversible condition characterized by myocardial akinesis due to catecholamine-mediated myocardial stunning. Acute intermittent porphyria (AIP) is associated with a rise in catecholamine, which could trigger TC. This study aims to evaluate patients with porphyria-triggered TC.

Methods

Data from the National Inpatient Sample (NIS) was used to study the prevalence rate and clinical outcome of porphyria-triggered TC among patients with TC.

Results

Overall, 32,500 cases were admitted between 2012 and 2016 with TC. The rates of smoking, hypertension, hyperlipidemia, and diabetes mellitus were 28%, 54%, 45%, and 23%, respectively. Six and three percent had cardiogenic shock and cardiac arrest, respectively. The overall inpatient mortality was 5.4%. Out of 32,500 patients with takotsubo cardiomyopathy, only three of these cases were found to have porphyria. Patients with porphyria were not significantly different in the baseline health characteristics from patients without porphyria. Additionally, there were no significant differences in the inpatient clinical outcomes between patients with porphyria vs. patients without porphyria.

Conclusion

TC triggered by porphyria is a rare disease. Patients with this disease have an excellent short-term prognosis. Beta-blocker medications might be effective in these patients to reduce the risk of recurrence. Further prospective studies are needed to test the effectiveness of beta-blocker in reducing the recurrence of TC.

## Introduction

Takotsubo cardiomyopathy (TC), also known as stress-induced cardiomyopathy, is a reversible condition characterized by apical akinesis resulting in apical ballooning [1, 2]. This cardiomyopathy was first described in Japan in the early 1990s. It was named after Japanese octopus traps with broad bases and narrow necks resembling the left ventricle in patients with TC [3]. The pathophysiology of TC remains unknown. However, TC is usually triggered by emotional stress, which is thought to cause catecholamine-mediated myocardial stunning in these patients [4-6].

The typical clinical symptoms of TC are chest pain and dyspnea, which mimic acute myocardial infarction. ST-segment elevation with a rise in cardiac enzymes may be seen in patients with TC but usually less than what is seen in patients with acute myocardial infarction [7, 8]. The inpatient shock and death rate is not significantly different between TC patients and acute myocardial infarction patients [9, 10]. Left ventricular function usually recovers within two to three months with a recurrence rate of approximately 11% [11-13].

Acute intermittent porphyria (AIP) is an autosomal dominant inherited disorder caused by a defect in the porphobilinogen-deaminase enzyme (also known as hydroxymethyl bilane synthase) [14, 15]. A defect in this enzyme, which involves the third step of the heme biosynthetic pathway, accumulates porphyrin precursors, porphobilinogen, and aminolevulinic acid [16]. Accumulating these porphyrin precursors leads to autonomic and peripheral neuropathy, which causes symptoms of acute porphyria attacks [17]. AIP can be associated with a rise in catecholamine [18], which could trigger TC. Only three case reports in the medical literature report patients with TC triggered by AIP [19-21].

This study aims to examine the prevalence rate of porphyria-triggered TC and describe these patients' baseline characteristics and clinical outcomes.

## Materials and methods

Study population 

The NIS data from the Healthcare Cost and Utilization Project (HCUP) were used in this study. The NIS is the largest nationally publicly available inpatient care database in the United States. This database was designed to evaluate inpatient costs, access, utilization, quality, and outcomes. The NIS contains baseline demographics and health characteristics, in-hospital procedures, and clinical outcomes for each patient. 

All patients admitted with TC between 2012 and 2016 were identified based on the International Statistical Classification of Diseases and Related Health Problems (ICD) code (TC: ICD9: 429.83/ ICD10: I51.81), n=32,500 patients. We then identified patients with porphyria (ICD9: 27.71/ ICD10: E80.21) among this group. Three cases were identified in the NIS with a diagnosis of TC and porphyria.

Clinical variables and outcomes

Baseline demographic characteristics included age, sex, year of admission, and hospital census region. Based on ICD9 and ICD10 codes, baseline health characteristics were identified, including smoking status, diabetes mellitus, hypertension, hyperlipidemia, ischemic heart disease, atrial fibrillation/flutter, chronic kidney disease, and sepsis. The inpatient death and length of stay were the primary outcomes. The secondary outcomes included cardiogenic shock, cardiac arrest, and the need for mechanical ventilation and circulatory support.

Statistical analysis

The baseline demographic and health characteristics were compared between the two groups, patients with porphyria and those without, using the Chi-squared and Wilcoxon tests for binary and continuous variables. Chi-squared test and Wilcoxon test were also used to test for differences in the clinical outcomes between the two groups. The statistical analyses were performed using SAS version 9.4 (SAS Institute Inc., Cary, North Carolina) and STATA version 16 (StataCorp LLC, College Station, Texas). All tests used an alpha of 0.05 as the probability for a type I error.

## Results

Overall, 32,500 cases were admitted between 2012 and 2016 with TC. The median age of these patients was 68 years. Eighty-five percent of these patients were women. The rates of smoking, hypertension, hyperlipidemia, and diabetes mellitus were 28%, 54%, 45%, and 23%, respectively. The rates of atrial flutter/fibrillation, chronic kidney disease, and sepsis were 20%, 13%, and 11%, respectively. Table 1 shows the baseline demographic and health characteristics of all patients with TC.

**Table 1:** Baseline demographic and clinical characteristics of patients with takotsubo cardiomyopathy, N=32,500

Characteristics	Total, %
Female	85
Age in years (median)	68
Admission year	
2012	15.6
2013	18
2014	19.7
2015	22.7
2016	24
Region	
Northeast	19.3
Midwest	25.7
South	32.8
West	22.2
Smoking	28.1
Hypertension	54.3
Hyperlipidemia	44.9
Diabetes mellitus	22.7
Atrial flutter/fibrillation	19.9
Chronic kidney disease	13.3
Sepsis	11

Regarding the inpatient clinical outcome, the median length of stay was four days. Nineteen and three percent of patients had acute kidney injury and cerebrovascular accident, respectively. Six and three percent had cardiogenic shock and cardiac arrest, respectively. Eighteen and two percent of these patients required mechanical ventilation and mechanical support, respectively. The overall inpatient mortality was 5.4%. Table 2 shows the inpatient clinical outcomes of all patients with TC.

**Table 2:** Inpatient clinical outcome of patients with Takotsubo cardiomyopathy.

Table 2. Inpatient clinical outcome of patients with Takotsubo cardiomyopathy.	
Inpatient Outcome	Total
Acute kidney injury	19.0
Cardiogenic Shock	5.7
Cardiac Arrest	3.17
Mechanical Support	1.68
Mechanical Ventilation	17.9
Cerebrovascular Accident	3.3
Length of stay in day, median	4
Inpatient Death	5.4

Out of 32,500 patients with takotsubo cardiomyopathy, only three of these cases were found to have porphyria. Patients with porphyria were not significantly different in the baseline health characteristics from patients without porphyria. Additionally, there were no significant differences in the inpatient clinical outcomes between patients with porphyria vs. patients without porphyria. 

## Discussion

This study found that TC triggered by porphyria is a rare disease, with an estimated incidence of 1 in 10,000 patients with TC. The study also found that these patients had a good prognosis, with no inpatient deaths. The exact mechanism of TC triggered by porphyria is unknown. However, it is thought to be caused by a rise in catecholamines in patients with acute porphyria. Another recently described theory is the dysregulated corticosteroid hormonal balance, which leads to a maladaptive catecholaminergic response in cardiac tissue and is another important TC determinant. Hence, both excess and deficiency of corticosteroids can lead to TC [22]. In animal models, the accumulation of porphyrin precursors significantly reduces the catecholamine uptake, which causes the accumulation of circulating catecholamines [18]. This increase in catecholamines could cause catecholamine-mediated myocardial stunning, leading to TC. Additionally, the clinical presentation of acute porphyria is associated with severe abdominal pain, which could be a physical stressor and a trigger for TC [23].

There is limited data on the treatment of TC triggered by porphyria. However, the available evidence suggests that IV hemin and beta-blockers may effectively treat this condition. IV hemin reduces the production of porphyrin precursors from bone marrow and liver, while beta-blockers block the effects of catecholamines.

In the medical literature, there are three case reports for patients with TC triggered by porphyria. The patients were successfully treated with IV hemin and beta-blockers in all three cases. The first case, published in 2016, involved a 44-year-old woman admitted with an acute porphyria episode and developed cardiogenic shock with multi-organ failure. Her echocardiogram revealed typical images of TC, and her cardiac catheterization showed no evidence of coronary obstruction. She was treated with IV hemin and beta-blockers, and her left ventricle (LV) function normalized within one month [21]. The second case, published in 2018, involved a 32-year-old woman who had frequent admissions for severe abdominal pain that was thought to be secondary to endometriosis. However, she developed TC with severe abdominal pain during her last admission. At that time, she had a porphyria workup and was diagnosed with acute intermittent porphyria. She was also treated with IV hemin and beta-blockers, and her LV function normalized within two weeks [20]. The third case, published in 2022, involved a 21-year-old woman with recurrent TC triggered by acute intermittent porphyria. She developed severe LV dysfunction with clinical features of heart failure. She was managed successfully with IV hemin and beta-blockers, and her LV function normalized after three weeks of discharge while she was on bisoprolol and losartan [19].

IV hemin has shown in previous cases that it effectively treats takotsubo cardiomyopathy triggered by porphyria with an excellent short-term prognosis. However, we expect these patients to have a high recurrence rate due to the underlying chronic disease, which could trigger Takotsubo cardiomyopathy. Upon discharge, patients should be managed with an angiotensin-converting enzyme (ACE) inhibitor or angiotensin receptor blocker (ARB), as studies have shown that these medications reduce mortality and recurrence [9, 24]. Although beta-blockers have not been shown to be beneficial upon discharge to minimize the risk of recurrence in general [9, 24], we expect that beta-blockers will be beneficial in patients who have takotsubo cardiomyopathy triggered by porphyria because these patients have a chronic increase in catecholamines due to porphyrin precursors [18].

## Conclusions

The findings of this study suggest that TC triggered by porphyria is a rare disease with a good prognosis when treated with IV hemin and beta-blockers. Beta-block medications might be effective in these patients to reduce the risk of recurrence. Further prospective studies are needed to test the effectiveness of beta-blocker in reducing the recurrence of takotsubo cardiomyopathy.
